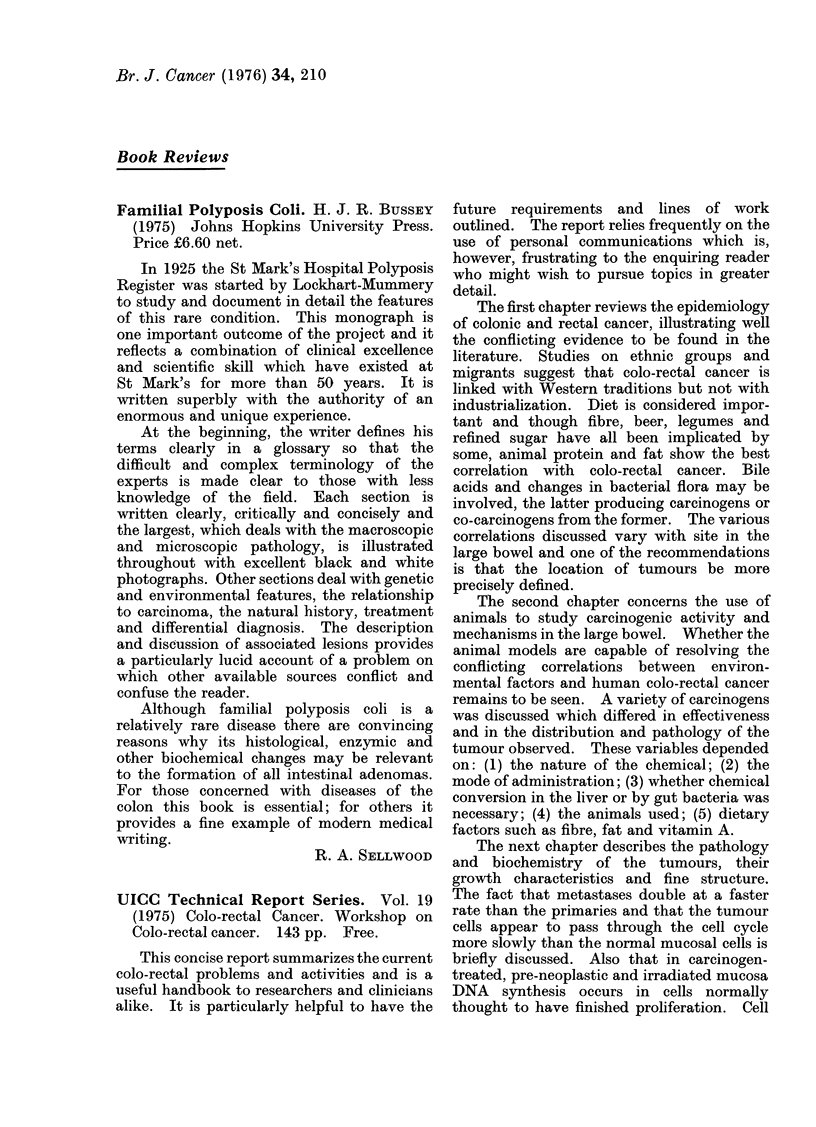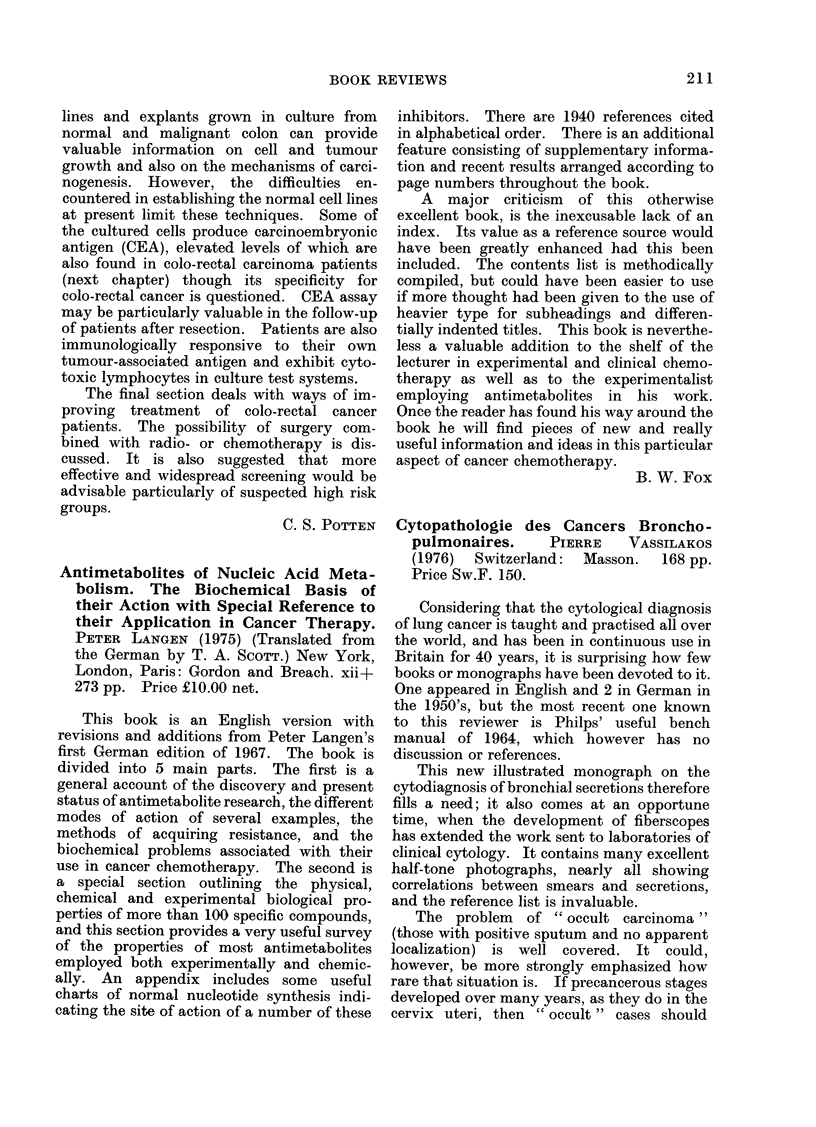# UICC Technical Report Series

**Published:** 1976-08

**Authors:** C. S. Potten


**UICC Technical Report Series.** Vol. 19 (1975) Colo-rectal Cancer. Workshop on Colo-rectal cancer. 143 pp. Free.

This concise report summarizes the current colo-rectal problems and activities and is a useful handbook to researchers and clinicians alike. It is particularly helpful to have the future requirements and lines of work outlined. The report relies frequently on the use of personal communications which is, however, frustrating to the enquiring reader who might wish to pursue topics in greater detail.

The first chapter reviews the epidemiology of colonic and rectal cancer, illustrating well the conflicting evidence to be found in the literature. Studies on ethnic groups and migrants suggest that colo-rectal cancer is linked with Western traditions but not with industrialization. Diet is considered important and though fibre, beer, legumes and refined sugar have all been implicated by some, animal protein and fat show the best correlation with colo-rectal cancer. Bile acids and changes in bacterial flora may be involved, the latter producing carcinogens or co-carcinogens from the former. The various correlations discussed vary with site in the large bowel and one of the recommendations is that the location of tumours be more precisely defined.

The second chapter concerns the use of animals to study carcinogenic activity and mechanisms in the large bowel. Whether the animal models are capable of resolving the conflicting correlations between environmental factors and human colo-rectal cancer remains to be seen. A variety of carcinogens was discussed which differed in effectiveness and in the distribution and pathology of the tumour observed. These variables depended on: (1) the nature of the chemical; (2) the mode of administration; (3) whether chemical conversion in the liver or by gut bacteria was necessary; (4) the animals used; (5) dietary factors such as fibre, fat and vitamin A.

The next chapter describes the pathology and biochemistry of the tumours, their growth characteristics and fine structure. The fact that metastases double at a faster rate than the primaries and that the tumour cells appear to pass through the cell cycle more slowly than the normal mucosal cells is briefly discussed. Also that in carcinogen-treated, pre-neoplastic and irradiated mucosa DNA synthesis occurs in cells normally thought to have finished proliferation. Cell lines and explants grown in culture from normal and malignant colon can provide valuable information on cell and tumour growth and also on the mechanisms of carcinogenesis. However, the difficulties encountered in establishing the normal cell lines at present limit these techniques. Some of the cultured cells produce carcinoembryonic antigen (CEA), elevated levels of which are also found in colo-rectal carcinoma patients (next chapter) though its specificity for colo-rectal cancer is questioned. CEA assay may be particularly valuable in the follow-up of patients after resection. Patients are also immunologically responsive to their own tumour-associated antigen and exhibit cytotoxic lymphocytes in culture test systems.

The final section deals with ways of improving treatment of colo-rectal cancer patients. The possibility of surgery combined with radio- or chemotherapy is discussed. It is also suggested that more effective and widespread screening would be advisable particularly of suspected high risk groups.

C. S. Potten